# Limits to the Evaluation of the Accuracy of Continuous Glucose Monitoring Systems by Clinical Trials

**DOI:** 10.3390/bios8020050

**Published:** 2018-05-18

**Authors:** Patrick Schrangl, Florian Reiterer, Lutz Heinemann, Guido Freckmann, Luigi del Re

**Affiliations:** 1 Institute for Design and Control of Mechatronical Systems, Johannes Kepler University Linz, 4040 Linz, Austria; patrick.schrangl@jku.at (P.S.); luigi.delre@jku.at (L.d.R.); 2 Science & Co, 41462 Neuss, Germany; lutz.heinemann@profil.com; 3 Institut für Diabetes-Technologie, Forschungs- und Entwicklungsgesellschaft mbH an der Universität Ulm, 89081 Ulm, Germany; guido.freckmann@idt-ulm.de

**Keywords:** blood glucose, diabetes, continuous glucose monitoring, quality of measurement

## Abstract

Systems for continuous glucose monitoring (CGM) are evolving quickly, and the data obtained are expected to become the basis for clinical decisions for many patients with diabetes in the near future. However, this requires that their analytical accuracy is sufficient. This accuracy is usually determined with clinical studies by comparing the data obtained by the given CGM system with blood glucose (BG) point measurements made with a so-called reference method. The latter is assumed to indicate the correct value of the target quantity. Unfortunately, due to the nature of the clinical trials and the approach used, such a comparison is subject to several effects which may lead to misleading results. While some reasons for the differences between the values obtained with CGM and BG point measurements are relatively well-known (e.g., measurement in different body compartments), others related to the clinical study protocols are less visible, but also quite important. In this review, we present a general picture of the topic as well as tools which allow to correct or at least to estimate the uncertainty of measures of CGM system performance.

## 1. Introduction

### 1.1. Background

Diabetes is a chronic disease with high prevalence and incidence, characterized by a malfunctioning of the physiologic glucose/insulin metabolism, mainly due to a lack of sufficient endogenous insulin secretion (type 1 diabetes, t1d) and/or to the loss of insulin sensitivity of the respective tissues in the human body (type 2 diabetes, t2d).

The standard therapy for t1d and later stages of t2d consists of replacing or adding insulin by subcutaneous administration to keep the blood glucose (BG) concentration within a range known to be best both for reducing the risk of developing long-term diabetes-related complications and avoiding acute complications such as hypoglycemic events.

Against this background, correct and rapid measurements or even predictions of BG values [1] are a key factor to ensuring a good clinical outcome. Unsurprisingly, a huge number of devices have been proposed to measure glucose, in particular systems for the self-monitoring of blood glucose (SMBG), which are usually based on enzymatic reactions with glucose in a small sample of capillary blood extracted from a finger tip (see [2] for an overview on the current market). A number of non-invasive glucose monitoring approaches have also been proposed, but none of these was able to reach true market maturity.

The main alternative to SMBG is continuous glucose monitoring (CGM), which is based on the measurement of the glucose concentration in the interstitial fluid around the tip of a needle-like glucose sensor inserted in the subcutaneous adipose tissue, mainly in the abdominal region. While a CGM sensor can remain in place for several days, a SMBG requires a small capillary blood sample each time (i.e., pinching one finger). As a consequence, CGM provides glucose measurements at a much higher sampling frequency (e.g., every 5 min) compared to SMBG (typically a few times a day).

Since the sampling time is considerably smaller than the time constants of the glucose metabolism, CGM helps users to avoid missing important events (in particular, hypoglycemic episodes), and even allows some prediction of impending critical situations. CGM systems can also be used to design novel algorithms for diabetes therapy, such as the artificial pancreas (AP). CGM has become increasingly popular over the past couple of years, and is expected to become even more widespread in the near future—that is, it might become the standard for glucose monitoring within 10 years (if costs can be reduced massively).

However, using CGM data as the primary basis for therapeutic decisions is not trivial. Indeed, CGM measures the glucose concentration in the interstitial fluid and not in the blood, as SMBG does. Of course, both glucose concentrations are closely related by a diffusion process, and CGM readings can be calibrated to correspond to the equivalent SMBG values under steady state values, but larger differences arise when glucose levels are rapidly changing, in which cases changes the BG values exhibit time differences on the order of 5 to 10 min [3].

### 1.2. Challenges of the Performance Assessment of CGM Systems

Ever since the early days of CGM technology development, the adequate performance assessment of CGM systems has been a question of concern (e.g., [4]). As with most sensors, an evaluation of the performance of CGM systems under repeatable conditions by so called in vitro tests is possible and widely done. However, it turns out that the results of in vitro tests do not reflect the real in vivo behavior of CGM systems for their intended use, as a number of patient-specific factors influence this massively. These cannot be reproduced under in vitro settings.

Therefore, the evaluation of CGM systems must be done by clinical trials. In these trials, CGM readings are compared to reference BG measurements obtained for the same patients at the same time. The key problem with this approach is that there is no practically feasible way to know the “real” glucose concentration in the interstitial tissue around the CGM sensor (in other words, to assess the “true” accuracy of the sensor in terms of the correct measurement of the glucose concentration it is exposed to). Therefore, performance assessment of CGM devices is usually done by comparison with SMBG point measurements (e.g., [5]).

There are a multitude of CGM performance measures, such as precision absolute relative deviation (PARD) [6] or the percentage of points in different areas of a continuous glucose error grid analysis (CG-EGA) [7] (or in the Clarke [8] or consensus error grid analysis [9]). However, over the past couple of years, an accuracy metric called the mean (sometimes median is used instead of mean; in this paper, we use the mean value) average relative deviation (MARD) has become popular, and is by far the measure used most often to characterize the analytical performance of CGM systems [5,10,11,12,13,14,15,16,17,18,19,20,21,22,23,24,25,26].

The MARD is based on the comparison between paired measurements of a given CGM system and a reference method for BG measurements which is implicitly assumed to be “true”. MARD is computed as the sample mean value of the absolute relative differences (ARDs) between the measurements of the CGM system and the corresponding BG values measured by the reference measurement device at the same time. Of course, ARDs can only be computed for those points in time when reference BG measurements are available.

This metric has many advantages, first among which is the simplicity, but the MARD can be misleading for several reasons (e.g., [27,28]), mainly related to the limits of the clinical study protocols—some of which, such as the small number of patients, are inherent. Indeed, an assessment based on clinical tests will reflect not only the “true” accuracy, but also several other factors independent of it and strongly related to the design of the clinical study. In fact, the enormous progress which has recently been made in improving the “true” accuracy of CGM devices is expected to make its assessment by clinical studies less significant, as the computed MARD value will reflect more the conditions under which it was determined rather than its true value.

Unsurprisingly, a number of studies with CGM systems have been published in which significantly different MARD values have been reported for the very same CGM system (e.g., compare the MARD values for the Abbott FreeStyle Libre published in [21,23,25]; see also Table 2 in [29]). Accuracy measured in clinical settings may be quite different from accuracy observed under at-home conditions [14,22].

This paper is concerned with detailing these effects and with providing either a way to improve the assessment (e.g., by correcting for some influencing factors—for this purpose a weighted MARD, the so-called WMARD, will be suggested) or least by providing a quantitative estimate of the impact of other factors, proposing a “quality index” which essentially mirrors the reliability of the estimated precision.

### 1.3. Structure and Operation of a CGM Sensor

Today, a multitude of technologies which allow for an estimation of the BG concentration at a high sampling rate are known and therefore enable patients and physicians to gain insight into the glucose dynamics in the human body. These technologies are usually summarized under the term “CGM”, and typically consist of three elements: a sensor is used to measure some physical quantity that is somehow related to the glucose concentration, a transmitter sends the electric signals measured by the sensor which are then post-processed (filtered, etc.) and stored on a receiver unit, which is equipped with a screen for displaying the measured data and visualizing the glucose dynamics. The main differences between different CGM systems are found in the technology used for the glucose measurement and the physical quantity that is measured. A concise overview of different CGM systems can be found in [30,31,32].

By far the most mature CGM technology is the minimally invasive technique of using a needle-shaped glucose sensor filament which is inserted through the skin into the subcutaneous tissue and which remains there for 7 to 14 days. The CGM measures the glucose concentration in the interstitial fluid surrounding the sensor filament—the so-called interstitial glucose (IG) concentration, which lags behind BG by several minutes. In this paper, the term “CGM” always refers to this type of technology for measuring the glucose concentration.

Usually, the current measured by the glucose sensor can be assumed to be an affine function of the glucose concentration in the interstitial fluid. This allows for a simple calibration procedure of the glucose sensor of the CGM system by using corresponding SMBG values [33,34]. However, sensor sensitivity (i.e., the change in measured current as a function of a change in the glucose concentration, in nA/(mg/dL)) is not always the same for one sensor design, but is influenced by many other factors including the sensor insertion site, the insertion technique, the production date, etc. Additionally, the sensor sensitivity after insertion is also not constant, but changes over time. This is mainly due to the reaction of the body to the local trauma induced by inserting the sensor filament into the subcutaneous tissue. The body’s immune system tries to close the local wound caused by the sensor and starts to encapsulate it, which typically results in a loss of sensor sensitivity over time. This process is commonly known as “biofouling”. The level of biofouling can be reduced by using a suitable biocompatible sensor membrane, but it can never be fully eliminated. Most of the change in sensor sensitivity typically happens within the first 24 h of sensor use (“run-in phase”), after which point the change in sensitivity over time is usually lower (see also [35]).

Because the change in sensor sensitivity over time not only depends strongly on the properties of the sensor, but also on those of the patient and the sensor insertion site, the CGM current signal must be (re-)calibrated in vivo several times during the sensor’s life time (typically one to two times a day). This is usually done using finger prick SMBG measurements (typically every 12 h) in order to obtain estimates of the sensor sensitivity. Over the years, a multitude of advanced methods for CGM calibration have been proposed (e.g., [35,36,37,38,39,40,41]).

## 2. Materials and Methods

### 2.1. Study Data

The analysis presented in this paper is based on data from a recent clinical study performed at the Institute of Diabetes Technology, Ulm, Germany [15]. The main goal of this clinical trial was to compare the performance of three CGM systems from three different manufacturers in a head-to-head study. During the trial, twelve subjects with t1d spent seven days under in-patient conditions. During this time, each of them wore six CGM systems in parallel: two FreeStyle Navigator^TM^ by Abbott Diabetes Care, Alameda, CA, USA (in the following referred to as CGM1), two Guardian^TM^ REAL-Time by Medtronic MiniMed, Northridge, CA, USA (in the following referred to as CGM2), and two DexCom^TM^ Seven^®^ Plus by DexCom, San Diego, CA, USA (in the following referred to as CGM3). It should be mentioned that all three systems were slightly outdated by the current standards at the time of writing. However, this should not affect the generalizability of the results presented in this paper.

The recording with all CGM systems started in the morning of the first day of the clinical trial. Each individual wore all CGM systems for a specified time until removal as recommended by the corresponding manufacturer. The individuals therefore ended up wearing CGM1 for five clinical days, CGM2 for six clinical days, and CGM3 for seven clinical days. All CGM systems were calibrated regularly according to the corresponding manufacturer recommendations. Calibrations were performed using SMBG point measurements with CGM2’s built-in BG meter as reference device (= capillary blood). During the entire study period, all CGM signals were recorded, together with regular BG measurements by means of SMBG (using the built-in BG monitoring meters of CGM2; at least one per hour during the daytime and one during the night). Furthermore, venous blood samples were taken at specified times in parallel to SMBG and were analyzed regarding the plasma glucose concentration by means of a laboratory glucose analyzer, YSI 2300 START Plus, Yellow Springs, OH, USA (YSI) [42].

In accordance with the recommendations from [43], on days 2 and 5 of the study, glucose excursions were induced by serving fast-absorbing meals with high carbohydrate content and by delaying the subcutaneous insulin injections by 15 min, leading to considerable temporary peaks in the BG profiles. Furthermore, the injected insulin doses were increased by 15% compared to the recommended values, often leading to a subsequent hypoglycemia.

In order to be able to exactly quantify the effects of the different influencing factors on the MARD performance measure, it is helpful to have reliable BG values available at any point in time. However, SMBG cannot be measured as frequently. Therefore, an estimate of the continuous BG profiles based on SMBG and CGM data was computed using a method proposed by [44].

### 2.2. Problem Statement

MARD is defined as

(1) $$\begin{array}{cc} ARD_{k} & =100\%\;\cdot \;\frac{\left|y_{CGM}\left(t_{k}\right)-y_{ref}\left(t_{k}\right)\right|}{y_{ref}\left(t_{k}\right)}, \end{array}$$

(2) $$\begin{array}{cc} MARD & =\frac{1}{N_{ref}}\sum_{k=1}^{N_{ref}}ARD_{k}, \end{array}$$

where $y_{CGM}$ is the value measured by the CGM system, $y_{ref}$ is the value measured by the reference measurement device at time $t_{k}$, where $t_{k},k\in \left\{1,2,\ldots ,N_{ref}\right\}$ are the times when reference measurements are available. In this paper, SMBG values are taken as reference (i.e., $y_{ref}$ corresponds to the capillary BG concentration). In other works, venous BG is used instead (e.g., [19]).

Computation of MARD according to Equation (2) is straightforward. Note, however, that all measured values are affected by errors. That is, the measured glucose values consist of the true IG (for CGM) and BG values (for SMBG) with some additive distortions, as follows:

(3) $$\begin{array}{cc} y_{CGM}\left(t_{k}\right) & =IG\left(t_{k}\right)+w_{CGM}\left(t_{k}\right), \end{array}$$

(4) $$\begin{array}{cc} y_{ref}\left(t_{k}\right) & =BG\left(t_{k}\right)+v_{ref}\left(t_{k}\right). \end{array}$$

Therefore, Equation (1) for calculating the *k*-th ARD value can be rewritten as

(5) $$\begin{array}{cc} ARD_{k} & =100\%\;\cdot \;\frac{\left|y_{CGM}\left(t_{k}\right)-y_{ref}\left(t_{k}\right)\right|}{y_{ref}\left(t_{k}\right)}=100\%\;\cdot \;\frac{\left|IG\left(t_{k}\right)+w_{CGM}\left(t_{k}\right)-BG\left(t_{k}\right)-v_{ref}\left(t_{k}\right)\right|}{BG\left(t_{k}\right)+v_{ref}\left(t_{k}\right)}, \end{array}$$

with $w_{CGM}$ the CGM measurement error and $v_{ref}$ the error in the reference measurements. As a consequence, ARD is a stochastic variable, and every MARD value computed with a limited number of samples will approximate the “true” one within some confidence limits.

The purpose of this paper is to understand the factors that determine the disturbances $w_{CGM}$ and $v_{ref}$ and to estimate their impact (together with the impact of the small number of samples) on the confidence of the MARD estimation.

### 2.3. Problem Analysis

The most important factors influencing the MARD value—besides the measurement accuracy of the glucose sensor itself—are either of physiological nature or related to the study protocol (see also [27,28]).

Among the physiological aspects, two are dominant. The so-called “biofouling” (i.e., tissue changes near the insertion point) leads to changes of the sensor sensitivity, especially in the first day. This change can partially be compensated by calibration (and/or a mean curve for the expected sensitivity change over time), but will regardless result in a difference between the CGM measurements and BG. Since the extent of this effect differs between different CGM devices, it is considered in this paper as part of the CGM performance and will not be further discussed in the following. The second physiological aspect is due to the fact that changes in the BG and IG do not occur simultaneously, but there is a time delay between the two compartments which is due to physiology.

The key factors related to the study design are the choice of the reference quantity to be used as proxy of the real BG, the number of samples, and their distribution.

#### 2.3.1. Time Delay

The most widely accepted model for BG-to-IG dynamics is the simple two-compartment diffusion model [45]:

(6) $$\begin{array}{c} \tau \frac{d{}^{}}{dt^{}}IG\left(t\right)=-IG\left(t\right)+BG\left(t\right) \end{array}$$

In this model, τ corresponds to the physiological time delay, which was reported to be roughly 11 min [46].

The fact that IG lags behind BG creates a certain difficulty for BG control (for cases where CGM is used for insulin dosing as in an AP system, see [47]), but also for CGM calibration [48]. Therefore, CGM systems should be calibrated during periods with relatively constant BG concentration. As far as MARD is concerned, time lag can be tackled by compensating for this lag by means of a prediction algorithm, using the predicted instead of the actually measured CGM signal [49]. This is part of many CGM processing algorithms.

**Figure 1:**
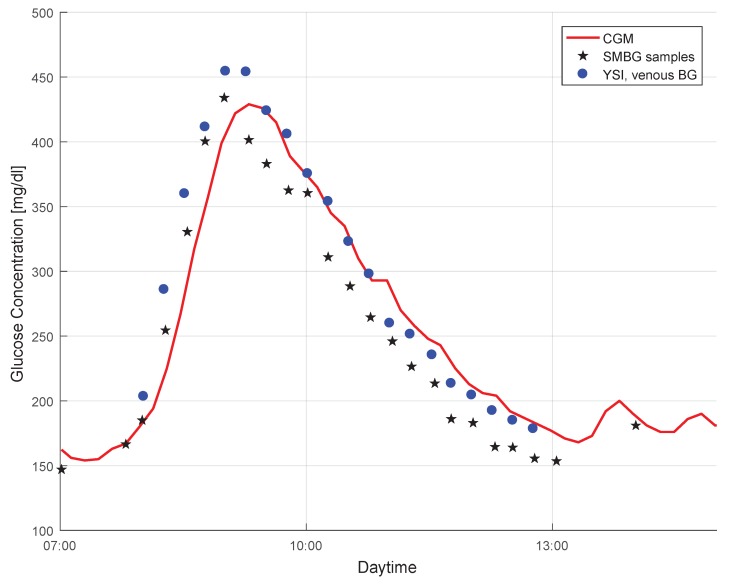
An illustration of the difference between the continuous glucose monitoring (CGM) profile, capillary blood glucose (BG) concentration measured with a BG meter (SMBG), and venous BG concentration measured with the YSI 2300 START Plus laboratory glucose analyser (YSI) (data from [15]).

The effect of the time lag between BG and IG is evident in Figure 1. The plot shows a data segment (red curve) in a CGM profile, and also the BG point measurements collected during the same time period. Both capillary BG measurements by means of SMBG (stars) as well as venous BG measurements by means of YSI (dots) are shown. The CGM signal lagged behind the SMBG measurements, but followed the BG profile well. It must be mentioned that the time delay between both signals was also caused by additional factors, such as time required for the measurement and filters used (see [49,50]).

#### 2.3.2. Choice of the Reference Method for Glucose Measurement

In Figure 1 a negative bias in the SMBG measurements can be seen as compared to the more accurate YSI values. Since the CGM data were calibrated with the SMBG measurement results, it also displays glucose values that were (most likely) lower than the actual BG values (see [51] for the clinical implications of this effect). Using YSI (or other laboratory glucose analyzers—e.g., [52]) data as a reference quantity for the performance assessment would therefore lead to somewhat different MARD values than using SMBG values, even though the CGM system is the same (see [53] for details on this effect). However, not only a bias in the reference measurements impacts MARD. Random measurement noise in the reference measurements also leads to significantly higher MARD values (see Section 3.2.1).

#### 2.3.3. Number of Samples

Since the measurement errors in the CGM and the reference measurements are stochastic in nature, MARD itself becomes a stochastic quantity. As can be expected, since MARD is an average of stochastic quantities, the amplitude of the stochastic portion of MARD will decrease with an increasing number of paired measurements. For a number of measurement points tending towards infinity, MARD is expected to converge towards a fixed value. On the other hand, the fewer paired measurements that are taken, the bigger will be the resulting uncertainty of MARD (see also Section 3.2.1).

#### 2.3.4. Distribution of Paired Measurement Points

The measurement errors of CGM are influenced by the glycemic range, with higher errors in the hypoglycemic and possibly also the hyperglycemic range [54]. Furthermore, the accuracy of BG meters that are used for the reference measurements is also significantly affected by the glucose concentration [55]. The amount of paired points measured in the different glycemic ranges will therefore have a direct impact on the resulting MARD value (see Section 3.2.2).

### 2.4. Composition of the Total MARD Error

So, the “true” sensor MARD (in the following referred to as $MARD_{0}$) would have to be obtained under the following conditions:

(7) $$MARD_{0}=E\left\{MARD\right\}=\underset{MARD_{0}}{\underset{︸}{\lim_{p\to p_{ref}}\underset{MARD_{1}}{\underset{︸}{\lim_{\tau \to 0}\underset{MARD_{2}}{\underset{︸}{\lim_{v_{ref}\to 0}\underset{MARD_{3}}{\underset{︸}{\lim_{N_{ref}\to \infty }\underset{MARD_{\mathrm{real}}}{\underset{︸}{\frac{100\%}{N_{ref}}\sum_{k=1}^{N_{ref}}\frac{\left|IG\left(t_{k}\right)+w_{CGM}\left(t_{k}\right)-BG\left(t_{k}\right)-v_{ref}\left(t_{k}\right)\right|}{BG\left(t_{k}\right)+v_{ref}\left(t_{k}\right)}}}}}}}}}}}$$

with $N_{ref}$, the number of reference measurements approaching infinity, $v_{ref}$, the error in the reference measurements going towards 0, the (physiological) time delay τ between BG and IG going towards 0, and the distribution of paired reference measurements *p* corresponding to some predefined standardized reference distribution $p_{ref}$. $MARD_{0}$ corresponds to the expected value of MARD under these ideal conditions. The intermediary steps where only some of these criteria are met will be referred to as $MARD_{1}$, $MARD_{2}$, and $MARD_{3}$, whereas the MARD obtained in the actual clinical trial will be denoted $MARD_{\mathrm{real}}$.

Under real-life conditions, the criteria necessary to obtain $MARD_{0}$ are impossible to meet. Deviations from those ideal conditions will result in MARD values that are significantly different from $MARD_{0}$. Whereas some of the factors influencing MARD only lead to a stochastic effect which results in an uncertainty in MARD (limited number of paired reference measurements, distribution of paired measurement points), others cause a significant increase in MARD (error in the reference measurement, physiological CGM time delays). In the following formula, it is shown how each of these factors leads to an increase in the uncertainty of MARD (expressed as the standard deviation of MARD, $\mathrm{SD}\left\{MARD\right\}$) and possibly also an increase in the expected value of MARD ($E\left\{MARD\right\}$):

(8) $$\begin{array}{c} MARD_{0}=E\left\{MARD_{1}\right\}\leq E\left\{MARD_{2}\right\}\leq E\left\{MARD_{3}\right\}\leq E\left\{MARD_{\mathrm{real}}\right\}, \end{array}$$

(9) $$\begin{array}{c} 0=\mathrm{SD}\left\{MARD_{0}\right\}\leq \mathrm{SD}\left\{MARD_{1}\right\}\leq \mathrm{SD}\left\{MARD_{2}\right\}\leq \mathrm{SD}\left\{MARD_{3}\right\}\leq \mathrm{SD}\left\{MARD_{\mathrm{real}}\right\}. \end{array}$$

**Figure 2:**
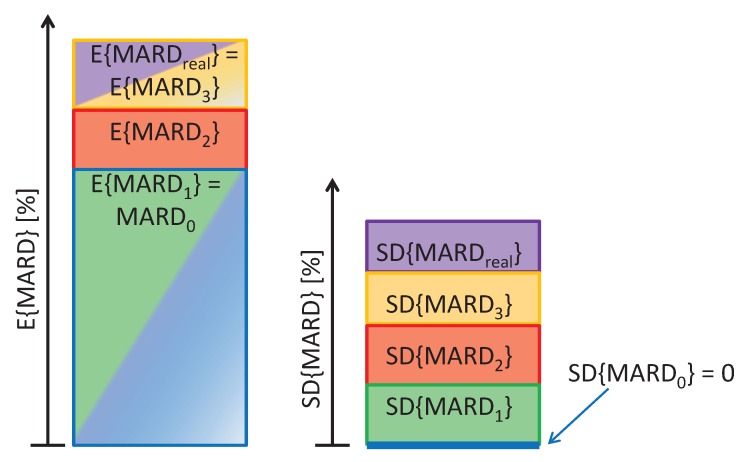
Effect of influencing factors on expected value and uncertainty (expressed through standard deviation) of mean average relative deviation (MARD).

Whereas each of the influencing factors is expected to increase $\mathrm{SD}\left\{MARD\right\}$, only the error in the reference measurements and the physiological CGM time delay lead to an increase in the expected value $E\left\{MARD\right\}$. This is also visualized in Figure 2.

## 3. Results and Discussion

### 3.1. Limits of the Perfect Glucose Sensor: Estimation of the Target Value under Perfect Conditions

As mentioned previously, there is a significant delay between IG and BG caused by the time needed for glucose to diffuse from one compartment to the other. So, even if a CGM system perfectly measures IG without any errors or additional time delays and the reference measurement device perfectly measures BG, still, due to dynamical changes in the glucose value, ARD values different from 0 would result. Consequently, MARD not only depends on the CGM system’s performance and the study design, but ARD values larger than 0 are also unavoidable due to physiological reasons.

It should be mentioned that by means of smart post-processing algorithms, the effect of CGM time delays can potentially be significantly reduced or almost eliminated (e.g., the smart sensor concept [37]). Modern CGM systems have a much smaller time delay than older devices, with values that are typically lower than the physiological time delay (e.g., [56,57,58]).

**Figure 3:**
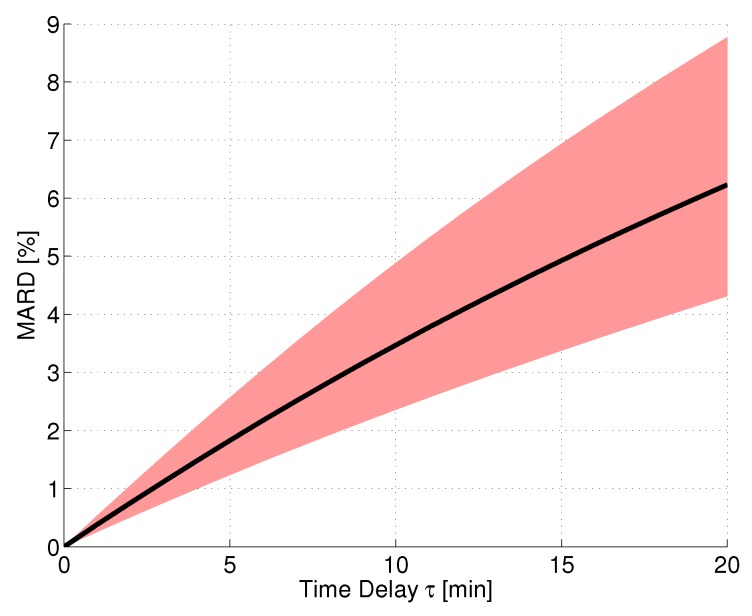
Effect of magnitude of time delay between IG and BG on MARD for a perfect CGM system tested under ideal conditions. Black line: mean MARD (mean over 12 patients from data); red area: possible MARDs based on different BG dynamics of patients (borders correspond to patients with min/max glycemic variability).

In order to simulate a CGM system performance assessment under ideal conditions, an in silico study was performed using the continuous BG traces calculated with the approach from [44] based on the clinical data from trial [15] (see Section 2). For this evaluation, it was assumed that the sensor measured IG without any consideration of measurement noise, bad CGM calibrations, or other effects. Furthermore, the reference measurements were assumed to correspond to the BG concentration without any measurement errors. Based on the continuous BG traces, the corresponding IG traces were calculated using the two-compartment diffusion model, Equation (6), with different values of τ. The MARD was then calculated using all points along the glucose trajectories, thus having a very high number of paired measurements. The resulting MARD values as a function of the time delay value τ can be seen in Figure 3. The black line shows the average MARD of all 12 patients of the dataset, whereas the red-colored area displays the variation of MARD values between patients (minimum and maximum MARD). The physiological time delay alone (roughly 11 min [46]) resulted in MARD values on the order of 4%. The MARD values were different for different patients, because of their difference in glycemic variability: A higher level of glycemic variability (i.e., also higher glucose rate-of-change, ROC) results in larger differences between BG and IG (see also [59] for this effect).

### 3.2. Testing CGM Sensors under Real Conditions

#### 3.2.1. Impact of Statistical Errors

In this subsection, only the effect of the number of reference measurements $N_{ref}$ and the error in the reference measurements $v_{ref}$ on MARD are studied. Parts of these results have already been presented in [28]. Comparisons are made with respect to $MARD_{2}$ (i.e., the MARD for $N_{ref}\to \infty$ and for $v_{ref}\to 0$). This value was estimated for the data from [15] by using the continuous BG traces calculated using the method from [44] for obtaining the reference measurements. It is assumed here that the BG distribution corresponded to the representative one ($p=p_{ref}$, see Section 3.2.2 for details). Furthermore, the effect of the CGM time delays are not considered separately in this subsection (see Section 3.1 for more details on this topic).

##### Effects in Terms of Confidence

In order to correctly use MARD values, the study-related uncertainty should be considered. The current subsection demonstrates how this this uncertainty of MARD can be estimated by means of confidence intervals (see [60] for the mathematical background). The confidence interval (CI) of MARD indicates the range around the computed value in which the real value will lie with a given probability. For example, a 95% CI means the range around the computed value within which the “true” MARD—the one which corresponds to the accuracy of the device—will lie, with a probability γ of 95%. In all following explanations and results, a CI γ=95% will be used. However, it should be noted that a CI of 95% is very common, but somewhat arbitrary. Using larger values of confidence would yield similar results as the ones presented in this subsection, albeit with wider intervals.

##### Effect of Number of Paired Measurements ($N_{ref}$)

A key factor which affects the estimation of MARD is the number of paired points used. As already stated, MARD is a stochastic quantity. With an increasing number of paired points, the stochastic portion of MARD tends towards 0, as already shown phenomenologically in [27].

For the results displayed here, the data from [15] and the estimates for the continuous BG traces (see Section 2) were used. The continuous BG traces allow a reference value to be assigned to each CGM measurement. If all these points are used, the best approximation of the “real” MARD in terms of number of paired points can be computed. Since the estimates of the continuous BG traces are used, the error in the reference measurement system can be neglected in these computations. The resulting MARD values for $N_{ref}\to \infty$ therefore corresponded to $MARD_{2}$ and were lower than the MARD values stated in [15]. For example, for CGM2 a $MARD_{2}$ of 10.1% was calculated, whereas in [15] a MARD of 12.1% is stated.

**Figure 4:**
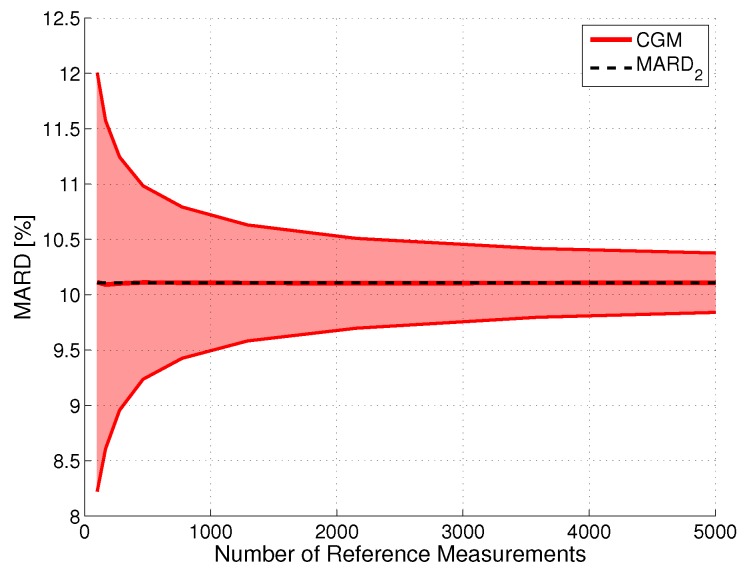
Impact of the number of paired points on the uncertainty of MARD for an illustrative CGM system (CGM2, the Guardian^TM^ REAL-Time unit): upper and lower bounds of the confidence interval (CI) with probability γ=0.95. The constant line represents the value to which it would converge for $N_{ref}$ towards infinity.

The effect of using fewer paired points was simulated as in [27] by randomly dropping some paired points in a Monte Carlo simulation. The results are shown in Figure 4 for CGM system CGM2 and a CI with γ=0.95. As expected, the width of the CI is proportional to $1/\sqrt{N_{ref}}$. This finding can also be justified from a mathematical point of view, because it is well-known that the variance of the sample mean decreases proportionally to $1/N_{ref}$; see [61,62]. The proportionality factor is CGM system-specific, with lower values for systems with a higher measurement performance. For the hypothetical case of the number of reference measurements approaching infinity, the CI would converge to one single point (i.e., $MARD_{2}$ in our case).

##### Effect of Errors in Reference Measurements ($v_{ref}$)

As explained previously, the limited accuracy of the reference measurements can have a strong impact on MARD, especially if BG meters and not laboratory analyzers are used during the performance assessment of CGM systems. In order to study this effect, Monte Carlo simulations using the computed continuous BG profiles for dataset [15] perturbed by random noise were performed. The error of the reference measurement device was assumed to be uncorrelated and Gaussian (mean error: 0%, accuracy error – expressed by the CI, γ=0.95: $err_{ref}$).

The effect of this error on the ARD can be computed by extending Equation (1) as follows:

(10) $$ARD_{i}=100\%\;\cdot \;\frac{\left|y_{CGM}\left(t_{i}\right)-y_{ref}\left(t_{i}\right)\;\cdot \;\left(1+e_{i}\right)\right|}{y_{ref}\left(t_{i}\right)\;\cdot \;\left(1+e_{i}\right)},\;e_{i}∼N\left(0,\frac{err_{ref}}{1.96}\right).$$

If the same reference measurement device is used for calibrating the CGM system and for drawing the paired reference measurements for the performance assessment (as is the case for the dataset used in this paper), only the stochastic part of the reference measurement error is expected to influence the MARD, but not the bias, since the same bias as in the reference measurements is also expected to be present in the CGM signals. Therefore, the effect of a bias in the reference measurements was not considered throughout this paper (see [53] for the effect on MARD of using different BG meters for calibrating the CGM and for drawing the paired reference measurements for the performance assessment, and see [51] for an overview of the clinical impact of a bias in the reference point measurements used for calibrating the CGM).

**Figure 5:**
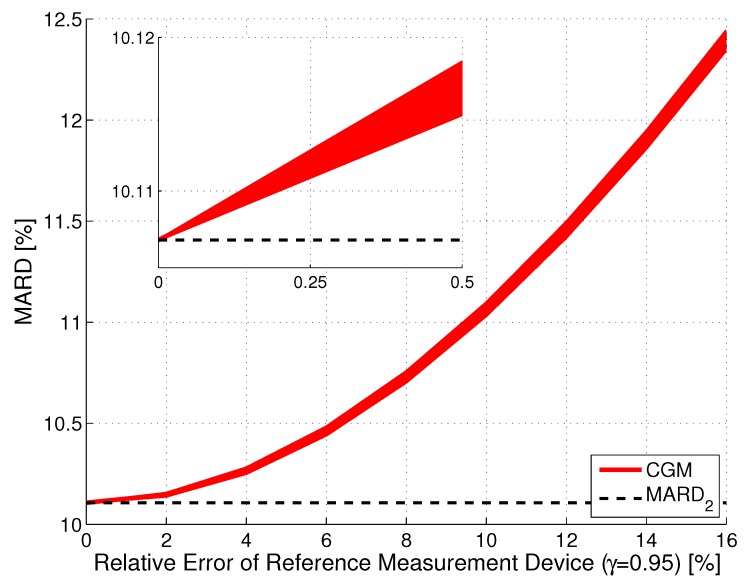
Effect of the relative error in the reference measurements on MARD for an illustrative CGM system (CGM2, CI, γ=0.95). The width of the line indicates the CI, the distance to the $MARD_{2}$ (black dashed line) reflects the error introduced by the error in the reference measurements.

Figure 5 shows the results of the Monte Carlo simulation for the illustrative CGM system CGM2. Notice that an increase of the error of the reference measurements did slightly increase the uncertainty (seen by the thickness of the red line), but primarily increased the value of MARD. This increase in MARD was proportional to $err_{ref}^{2}$, with a proportionality constant that depends on the value of $MARD_{2}$, with larger values of $MARD_{2}$ leading to a smaller proportionality constant. That is, the error in the reference measurements is more important for CGM sensors with a high measurement performance. Roughly speaking, the MARD computed from a clinical study is the sum of the MARD of the CGM system and of the MARD of the reference system. With the continuous improvement of CGM systems, a MARD value computed from the data of a clinical study in which SMBG devices were used as reference may turn out to be more of an evaluation of the accuracy of the SMBG device rather than of the CGM system. It should furthermore be noticed that the results shown in Figure 5 were obtained using the entire vector of paired points. For a lower number of reference points, this width would be larger.

##### Combined Effect of Number and Uncertainty of Reference Measurements ($N_{ref}$ and $v_{ref}$)

**Figure 6:**
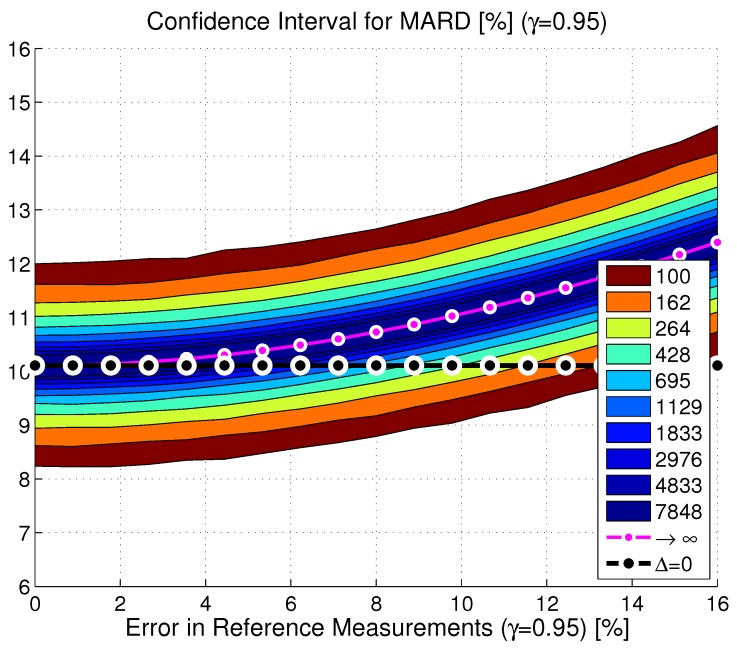
Confidence region of the MARD on the *y*-axis depending on the number of paired measurement points (see color code in the insert) and accuracy of the reference measurement system on the *x*-axis MARD for an illustrative CGM device (CGM2, CI, γ=0.95; black line: $MARD_{2}$, purple line: MARD for number of paired points towards infinity).

In practice, both effects discussed so far (the limited number of paired points and the limited accuracy of the reference measurements) appear jointly. This has been tested in Monte Carlo simulations using the dataset from [15] and the estimates of the continuous BG traces. An example for the resulting distribution of MARDs as a function of both quantities is shown in Figure 6 for CGM2. Analyses have shown that the MARD values in each point $\left(N_{ref,i},v_{ref,j}\right)$ followed a Gaussian distribution with mean value $MARD_{2}+\Delta_{MARD}$ and standard deviation $\sigma_{MARD}$. Based on an analysis of the entire dataset, it was found that offset $\Delta_{MARD}$ and standard deviation $\sigma_{MARD}$ could be computed according to the following formulas:

(11) $$\begin{array}{cc} \Delta_{MARD}\;\left[\%\right] & =a\;\cdot \;{\left(err_{ref}\;\left[\%\right]\right)}^{2}, \end{array}$$

(12) $$\begin{array}{cc} \sigma_{MARD}\;\left[\%\right] & =\frac{b+c\;\cdot \;(err_{ref}\;\left[\%\right])}{\sqrt{N_{ref}}}, \end{array}$$

(13) $$\begin{array}{c} a\;[{\%}^{-1}]=0.01244-0.00037\;\cdot \;MARD_{2}\;\left[\%\right] \end{array}$$

(14) $$\begin{array}{c} b\;[\%]=51.6178-7.7553\;\cdot \;MARD_{2}\;\left[\%\right]+0.3569\;\cdot \;{\left(MARD_{2}\;\left[\%\right]\right)}^{2}, \end{array}$$

(15) $$\begin{array}{c} c\;[{\%}^{-1}]=0.0037. \end{array}$$

It should be noted that the correlations in Equations (13) to (15) were derived for a $MARD_{2}$ range between 10% and 16%. For $MARD_{2}$ values outside this range, the values might not be appropriate.

##### Tackling MARD Uncertainty: Giving Bounds for MARD

**Figure 7:**
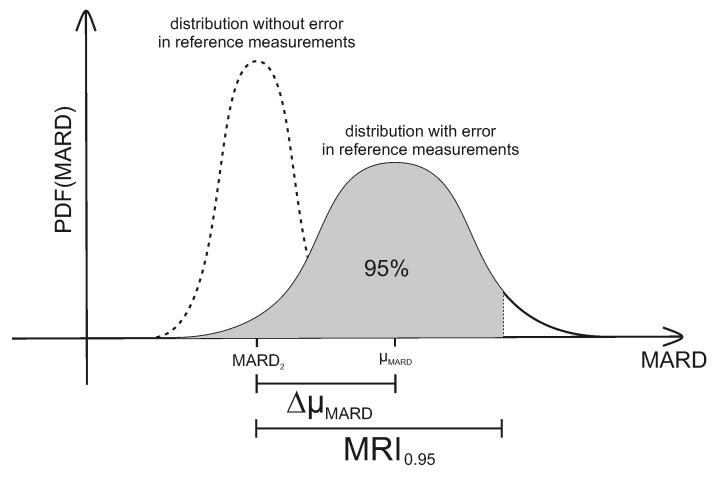
Combined effect of number of paired measurements and accuracy of the reference measurements on the uncertainty of MARD, here expressed by MARD reliability index (MRI). PDF: probability density function.

$N_{ref}$ and $v_{ref}$ are the main variables that can be easily controlled in the study design phase of clinical trials designed for assessing CGM performance. As can be seen earlier in this subsection, they have a huge impact on the reliability of the resulting MARD values. Therefore, $N_{ref}$ and $v_{ref}$ should be controlled in order to obtain MARD values that actually reflect CGM performance. In order to facilitate this, the so-called MARD reliability index (MRI) was introduced in [28]. The MRI is defined as the width of the symmetrical CI around $MARD_{2}$ (in %) that includes γ=95% of MARD values:

(16) $$\begin{array}{c} 0.95=\int_{MARD_{2}-MRI}^{MARD_{2}+MRI}p(MARD)\;dMARD, \end{array}$$

with p(MARD) the probability density function (PDF) of MARD values. MRI thus corresponds to an upper bound for the estimate of error in MARD as from a clinical trial (taking into account the effect of number and uncertainty of reference measurements). An illustration of MRI can also be seen in Figure 7 below. For the case of a very high accuracy of the reference measurement system, the probability of obtaining a given value of MARD is centered around $MARD_{2}$—the value corresponding to a very high number of measurements. This is shown on the left side of Figure 7. Increasing the number of paired points will narrow the PDF. An infinite number of paired points would of course deliver exactly $MARD_{2}$. Adding the effect of the error of the reference system leads to the solid curve on the right side of Figure 7. Note that this curve is no longer centered around $MARD_{2}$, but is displaced by the average value of the error of the reference system towards higher MARD values ($\mu_{MARD}$ in Figure 7).

**Figure 8:**
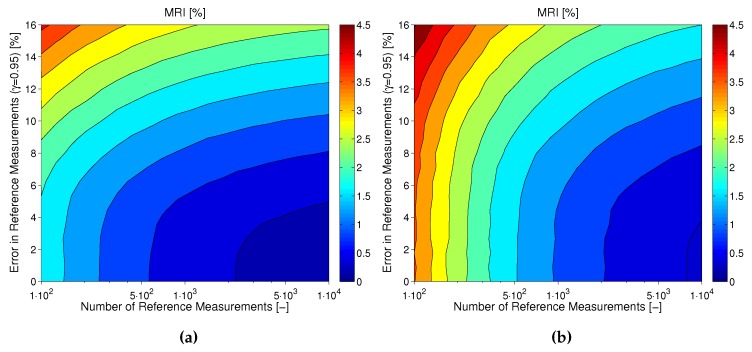
Comparison in terms of MRI for a CGM with (**a**) relatively good performance (CGM2) vs. (**b**) a CGM with worse performance (CGM3).

As explained earlier in this subsection, the uncertainty in MARD not only depends on $N_{ref}$ and $v_{ref}$, but is also influenced by the CGM performance itself. This also becomes evident for the results shown in Figure 8. In this figure, the MRI is shown for CGM2 (plot A) and CGM3 (plot B) of dataset [15] as computed based on the results of the Monte Carlo simulations presented earlier in this subsection. It can be seen how the effects of number and uncertainty of reference measurements look different for the two devices: whereas for CGM2 (with a relatively good measurement performance) the choice of the reference method is very important, for CGM3 (with an inferior measurement performance) a sufficiently high number of paired points is more important.

In order to determine the value of MRI as a function of $N_{ref}$ and $v_{ref}$, several plots like the ones in Figure 8 are supplied in [28], each for a different value of $MARD_{2}$. Alternatively, MRI can also be computed by numerically solving the following equation:

(17) $$\begin{array}{c} 0.95=\frac{1}{2}\;\cdot \;\left[\mathrm{erf}\left(\frac{MRI-\Delta_{MARD}}{\sqrt{2\;\cdot \;\sigma_{MARD}^{2}}}\right)-\mathrm{erf}\left(\frac{-MRI-\Delta_{MARD}}{\sqrt{2\;\cdot \;\sigma_{MARD}^{2}}}\right)\right], \end{array}$$

with $\Delta_{MARD}$ and $\sigma_{MARD}$ computed as a function of $N_{ref}$, $v_{ref}$, and $MARD_{2}$ according to Equations (11)–(15).

#### 3.2.2. CGM System Performance and BG Range

##### CGM System Performance Differs between BG Ranges

**Figure 9:**
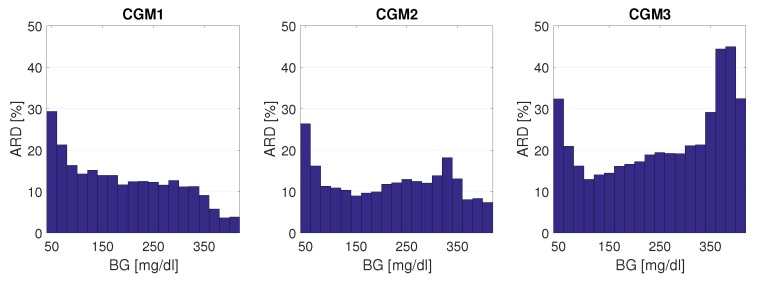
Absolute relative difference (ARD) as a function of BG range, from 40 mg/dL to 420 mg/dL. Bin width: 20 mg/dL. ARD: average over bin.

It was shown in [27] that, besides the number and accuracy of the reference measurements, the distribution function of the reference measurements also has a huge impact on the calculated MARD. As already mentioned, this is because the CGM system accuracy and SMBG accuracy depend on the BG range, with typically a lower accuracy in the hypoglycemic range (e.g., [54,55]). The effect of the BG range on CGM system performance can also be seen by the separate MARD values given in [15] for the hypoglycemic range, the hyperglycemic range, and the safe target range (euglycemic range). In order to better illustrate this effect, the relationship between ARD and BG values is shown in more detail for the three CGM devices from dataset [15] as a histogram plot in Figure 9 below. It can be seen that for CGM1 the ARD values were maximum in the hypoglycemic range and decreased continuously with increasing BG values, whereas for CGM2 and CGM3 there was a minimum in the euglycemic range and another increase when changing to the hyperglycemic range.

Additionally, aside from the distribution of paired measurement points as a function of BG range, the distribution of points in time might also have an impact on MARD values. For many CGM systems, ARDs are higher on the first days of use of the CGM system (e.g., [24]), as well as possibly towards the end of intended sensor lifetime (e.g., [15]). Furthermore, it is known that the estimate of the CGM sensitivity is best at the time of a calibration measurement, whereas afterwards the mismatch between estimated and true sensor sensitivity starts to increase over time [56], possibly leading to higher ARD values. However, by defining a uniform sampling in time of the paired measurement points in the study protocol, this effect should be minor. Therefore, it is not further considered in this paper.

##### Correcting for the Distribution of Points

In order to make the values of MARD comparable between studies, the distribution of the BG values in these studies should ideally be the same. Therefore, it is useful to define some kind of reference distribution, which serves as a standard reference for different clinical studies. From a clinical point of view, it is useful to define this reference distribution not arbitrarily, but in a way that is physiologically meaningful or typical for patients. Studies with a large number of patients have shown that the measurement data of the average patient is log-normally distributed. Therefore, it is proposed here to compute MARD using data that follow a well-defined reference BG distribution which is log-normally distributed. However, in practice, the obtained data from a real clinical trial will not have exactly this reference distribution. Therefore, a procedure is proposed in the current section that counteracts deviations from the reference distribution, which corrects the MARD value by weighing the data. The result of this computation is a weighted MARD (WMARD), which gives better comparability than MARD for results from different clinical trials, even if the distribution of BG values differs between these studies.

The WMARD can be defined by

(18) $$\begin{array}{c} WMARD=\frac{1}{W}\sum_{i=1}^{N}w_{i}\frac{\left|x_{i}-r_{i}\right|}{\left|r_{i}\right|}, \end{array}$$

with weights $w_{i}$ and $W=\sum_{i=1}^{N}w_{i}$. Here, $x_{i}$ is the measured CGM value and $r_{i}$ is the measured SMBG reference value. In order to shape the actual distribution p(r) of BG values towards the well-defined reference distribution $p_{ref}\left(r\right)$, the weights $w_{i}$ need to be computed as

(19) $$\begin{array}{c} w_{i}=\frac{p_{ref}\left(r_{i}\right)}{p(r_{i})}. \end{array}$$

**Figure 10:**
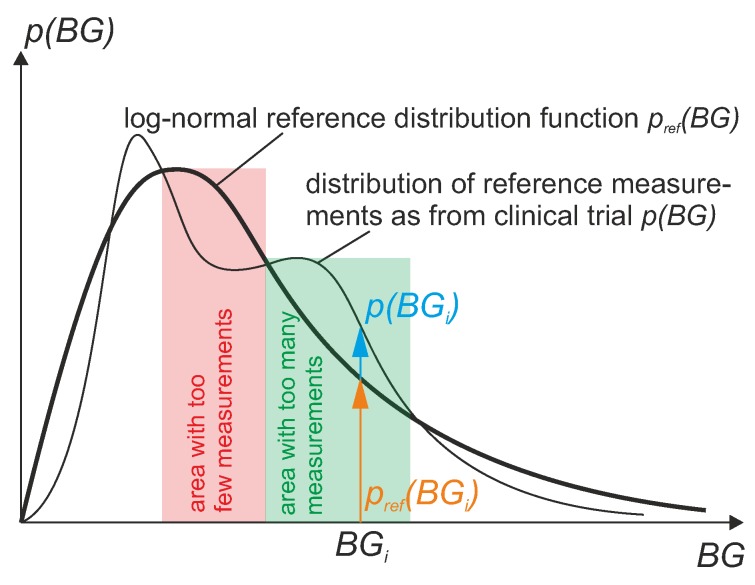
How to calculate WMARD based on the distribution *p* of reference BG values from clinical trial and reference distribution $p_{ref}$.

This is also illustrated in Figure 10.

For the definition of the reference distribution, it is proposed here to use a log-normally distributed function. For comparability of MARD between studies, the specific distribution does not need to be log-normally distributed; a fixed arbitrary distribution would be sufficient. However, from a clinical point of view, it is meaningful to normalize towards a typical distribution which is representative for an average patient, in order to avoid misleading MARD values. Therefore it is suggested to use the following parametrization of the reference distribution, which is based on data from [15]:

(20) $$\begin{array}{c} p_{ref}\left(x\right)=\frac{1}{k}\frac{1}{\sqrt{2\pi }\sigma x}\exp\left(-\frac{{\left(\ln\left(x\right)-\mu \right)}^{2}}{2\sigma^{2}}\right),\;0\leq x\leq u, \end{array}$$

with the following parameters:

(21) $$\begin{array}{cc} \mu & =4.9165, \end{array}$$

(22) $$\begin{array}{cc} \sigma & =0.3832, \end{array}$$

(23) $$\begin{array}{cc} u & =450\mathrm{mg}/\mathrm{dL}, \end{array}$$

(24) $$\begin{array}{cc} k & =\int_{0}^{u}\frac{1}{\sqrt{2\pi }\sigma x}\exp\left(-\frac{{\left(\ln\left(x\right)-\mu \right)}^{2}}{2\sigma^{2}}\right)dx. \end{array}$$

**Figure 11:**
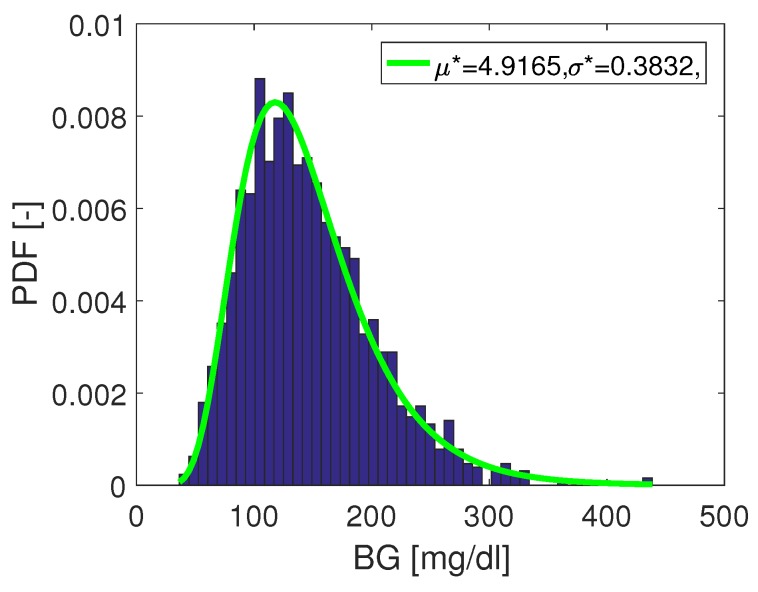
Reference distribution: as from data in trial by [15].

This distribution is also shown in Figure 11.

Another fact that needs to be discussed is that the actual BG distribution of the clinical trial must be estimated from the data in order to compute the weights. Since the WMARD also depends on the estimate p(BG) of this distribution, and p(BG) depends on the way in which it is estimated, it is also useful to specify how *p* should be estimated in order to improve comparability. Several ways of estimating variable distribution based on data can be found in the literature [63], the most popular being:

- histogram-based estimation, and
- kernel density estimation.

The simplest and most straightforward approach to distribution estimation is using a histogram. However, this approach has several well-known drawbacks that can influence the distribution estimate and therefore MARD significantly, such as:

- The *bin size* needs to be chosen wisely (according to number of data points).
- The *histogram shape* is substantially dependent on the position of the bin centers.

Due to these shortcomings, another approach called kernel density estimation (KDE) has been proposed [64], which is especially useful for estimating smooth, continuous distributions (as is also the case for a log-normal distribution). In order to reduce the degrees of freedom when using KDE to a minimum (to avoid different parameterizations for different studies that once again reduce comparability), it is suggested to use the following empirically-obtained parameters for the KDE:

- use of normally-distributed (Gaussian) kernel functions.
- bandwidth (standard deviation) of σ=10mg/dL.

**Figure 12:**
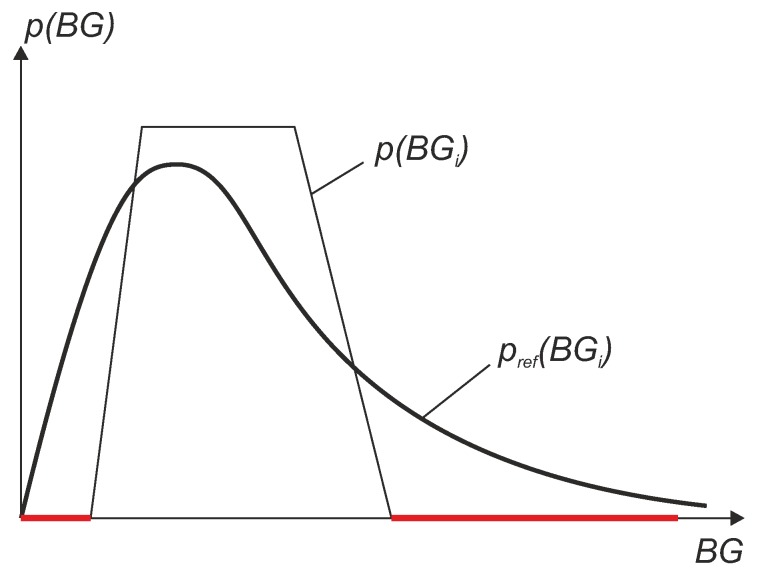
Illustration of a problem occurring if not all BG ranges are covered in the clinical data. Red lines represent regions with no data.

Note that for the computation of the weights $w_{i}$, a division by $p(r_{i})$ needs to be performed, which leads to a problem when this value is 0. This can be the case when in some BG regions no BG reference values have been measured within the clinical trial. The situation is illustrated in Figure 12, where the red lines represent regions with no data. In other words, if some BG regions are lacking data or have only a small number of points, a reconstruction is not possible or not reliable due to very high weights $w_{i}$ which result in a high uncertainty. This leads to an inaccurate value of the WMARD. To avoid this problem, it is suggested that in the study design it must be guaranteed that a sufficiently high amount of data points exist in each region. From empirical observation, the following suggestions are proposed:

- Between 70 mg/dL and 350 mg/dL in the measurement data of the reference sensor:
  - no gap larger than 10 mg/dL should exist in the data between 70 mg/dL and 200 mg/dL.
  - no gap larger than 20 mg/dL should exist in the data between 200 mg/dL and 350 mg/dL.
- more than 1% (and at least two points) of the data points exist in the hypoglycemic region below 70 mg/dL.
- more than 0.5% (and at least two points) of the data points exist in the hyperglycemic region above 350 mg/dL.

In order to show the effectiveness of WMARD compared to MARD when dealing with different study distributions, a simulation example was set up based on the data of [15]. Monte Carlo simulations were used in order to show that WMARD is very insensitive to the distribution of BG points (as long as all BG ranges are covered with a sufficiently high number of points), whereas MARD for the same sensor can differ significantly because of differences in distribution of BG points. The simulation study was performed as follows:

- The complete data set (*N* data pairs) of all sensors was used to select a subset with N/2 data points randomly within a Monte Carlo experiment (with 5000 repetitions).
- In each experiment, the MARD and WMARD were computed using the subset. The simulation was carried out for two cases:
  - the subset was selected in such a way that the reference data had a log-normal distribution, and
  - the subset was selected in such a way that the reference sensor data had a uniform-like distribution.
- The “true” MARD value (denoted $MARD^{*}$) was computed using the complete data set with *N* data pairs and this resulted in the value $MARD^{*}=16.1788\%$. It can be seen from the results that WMARD was hardly affected by the distribution of the paired points, whereas for the standard MARD, significant differences occurred for the different distribution functions.

**Figure 13:**
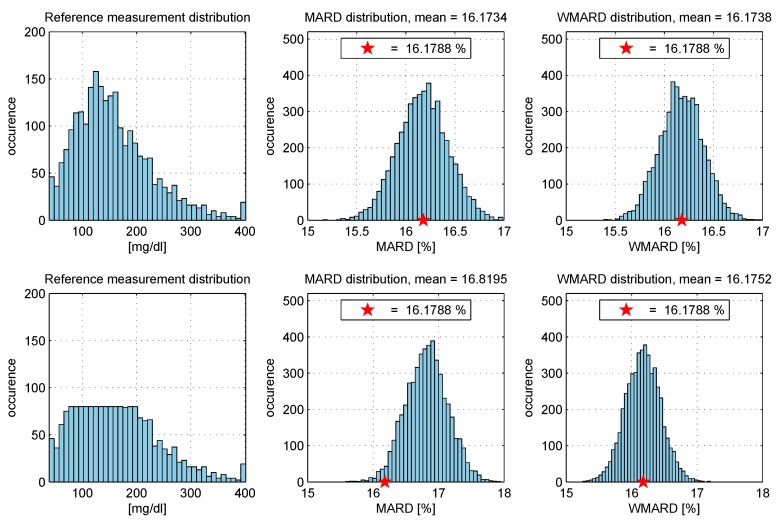
Simulation study: comparison between MARD and weighted MARD (WMARD) distributions when using different BG distributions.

The results are shown in Figure 13 and Table 1.

## 4. Conclusions

Assessing the performance of CGM systems is not a straightforward task. Nevertheless, it is very important to be able to draw conclusions regarding the performance of specific CGM systems. If physicians must choose between different alternatives, they want to recommend the best option for a CGM system to their patients. Similarly, patients themselves want to be able to take measurement performance into account when deciding on which CGM system to use. On a larger scale, health care insurance agencies need to make decisions about which CGM systems to reimburse and which ones not, and for doing so need to consider CGM system performance as one of the most important aspects. Aside from this economic dimension, CGM system performance is of course also important for other decisions that are more clinical in nature. For example, regulators (and manufacturers) need to determine whether or not a CGM system is suitable for non-adjunct use (i.e., to use the CGM system data also for insulin dosing decisions).

MARD is the most widely-used measure to assess the measurement quality of CGM systems. Its main advantage is its simplicity, since it condenses the entire complex information about CGM system measurement performance in one single number, which makes it easy to grasp. However, condensing this into one single number does not happen without any loss of information, and therefore the interpretation of MARD becomes more difficult than it seems at a first glance. It is important to understand that the MARD value not only depends on the quality of the CGM system, but is also influenced significantly by the clinical protocol of the trial during which the CGM system performance was assessed.

This paper presents a way to estimate the uncertainty in MARD stemming from the impact of the clinical protocol. The MRI introduced for this purpose offers a simple way to estimate how reliable published MARD values are. Again, it is a single number, like MARD, but conveys complementary information which is very important to avoid comparing things that cannot be practically compared. Additionally, this paper introduces the WMARD as a tool to minimize the impact of the distribution of paired measurement points (percentage in hypoglycemic, hyperglycemic, euglycemic range) on MARD. It does so by associating weights with each ARD value in order to end up with a MARD that is representative of a pre-defined reference distribution. WMARD thus partially eliminates the effect of the distribution of the paired measurement points.

The tools presented in this paper help with the interpretation of clinical trial results in terms of MARD, but of course, they do not eliminate the need for a unified clinical trial design for the assessment of CGM performance. It would be highly desirable to have a guideline for clinical studies for the assessment of CGM system performance which would be followed more frequently than guideline POCT05-A [43]. Additionally, it should not be forgotten that MARD does not reflect the very reason for the existence of CGM systems (i.e., frequent measurements). MARD thus needs to be complemented by other quantities designed for this purpose, such as PARD [6].

Regarding the clinical trial design and the analysis of published data concerning the measurement performance of CGM systems, the following conclusions can be drawn based on the data presented in this paper, especially if MARD is to be used as a performance measure:

- The number of paired measurements should be appropriately high in order to reduce the uncertainty in the average results.
- The accuracy of the reference measurement device should be significantly higher than the accuracy of the CGM system. This can be done by using either highly accurate laboratory glucose analyzers for the assessment of venous BG or a high-quality BG meter for assessing the capillary BG concentration. Since modern BG meters are factory calibrated in order display a value that is indicative of venous BG, it should not actually matter whether venous or capillary BG is used as reference quantity (in the case of a comparable accuracy, of course). However, it must be considered that BG meters might have a poor measurement performance in the case of, for example, hypoxemia or anemia. This should be taken into account in the inclusion and exclusion criteria when recruiting the study population for the clinical trial.
- The same reference measurement device should be used for both calibrating the CGM system and drawing the reference BG measurements.
- An overall MARD should never be the only source for interpretation of CGM accuracy. Instead, an overall MARD should be analyzed together with additional information, such as MARD values for different glucose ranges, as well as for different days of sensor use, distribution of MARD values over all analyzed CGM systems in the trial, percentage of large measurement errors, etc.
- If the performance of two CGM systems of different manufacturers have to be compared, this should ideally be done based on data from a head-to-head assessment. In case such data is not available, it would at least be recommendable to compare WMARD values from different studies and to take the MRI into account as well.

## Figures and Tables

**Table 1:** Simulation study: Comparison of mean values of MARD and WMARD for the two distributions.

Distribution	Mean Value of	
	MARD	WMARD
Log-normal	16.1734%	16.1738%
Uniform-like	16.8195%	16.1752%

## Abbreviations

The following abbreviations are used in this manuscript:

AP	artificial pancreas
ARD	absolute relative difference
BG	blood glucose
CG-EGA	continuous glucose error grid analysis
CGM	continuous glucose monitoring
CI	confidence interval
IG	interstitial glucose
KDE	kernel density estimation
MARD	mean absolute relative deviation
MD	medical doctor
MRI	MARD Reliability Index
PARD	precision absolute relative deviation
PDF	probability density function
ROC	rate-of-change
SMBG	self monitoring of blood glucose
t1d	type 1 diabetes
t2d	type 2 diabetes
WMARD	weighted MARD

## References

[1] Kirchsteiger H., Jørgensen J.B., Renard E., del Re L. (2016). Prediction Methods for Blood Glucose Concentration: Design, Use and Evaluation.

[2] Freckmann G., Schmid C., Ruhland K., Baumstark A., Haug C. (2012). System Accuracy Evaluation of 43 Blood Glucose Monitoring Systems for Self-Monitoring of Blood Glucose according to DIN EN ISO 15197. J. Diabetes Sci. Technol..

[3] Basu A., Dube S., Veettil S., Slama M., Kudva Y.C., Peyser T., Carter R.E., Cobelli C., Basu R. (2015). Time Lag of Glucose From Intravascular to Interstitial Compartment in Type 1 Diabetes. J. Diabetes Sci. Technol..

[4] Feldman B., Brazg R., Schwartz S., Weinstein R. (2003). A continuous glucose sensor based on Wired Enzyme^TM^ technology-Results from a 3-day trial in patients with type 1 diabetes. Diabetes Technol. Ther..

[5] Zschornack E., Schmid C., Pleus S., Link M., Klötzer H.M., Obermaier K., Schoemaker M., Strasser M., Frisch G., Schmelzeisen-Redeker G., Haug C., Freckmann G. (2013). Evaluation of the Performance of a Novel System for Continuous Glucose Monitoring. J. Diabetes Sci. Technol..

[6] Obermaier K., Schmelzeisen-Redeker G., Schoemaker M., Klötzer H.M., Kirchsteiger H., Eikmeier H., del Re L. (2013). Performance Evaluations of Continuous Glucose Monitoring Systems: Precision Absolute Relative Deviation is Part of the Assessment. J. Diabetes Sci. Technol..

[7] Kovatchev B., Cox D.J., Gonder-Frederick L.A., Clarke W.L. (2004). Evaluating the Accuracy of Continuous Glucose-Monitoring Sensors. Diabetes Care.

[8] Clarke W.L., Cox D., Gonder-Frederick L.A., Carter W., Pohl S.L. (1987). Evaluating Clinical Accuracy of Systems for Self-Monitoring of Blood Glucose. Diabetes Care.

[9] Parkes J.L., Slatin S.L., Pardo S., Ginsberg B.H. (2000). A new consensus error grid to evaluate the clinical significance of inaccuracies in the measurement of blood glucose. Diabetes Care.

[10] Weinstein R.L., Schwartz S.L., Brazg R.L., Bugler J.R., Peyser T.A., McGarraugh G.V. (2007). Accuracy of the 5-day FreeStyle navigator continuous glucose monitoring system. Diabetes Care.

[11] Kovatchev B., Anderson S., Heinemann L., Clarke W. (2008). Comparison of the Numerical and Clinical Accuracy of Four Continuous Glucose Monitors. Diabetes Care.

[12] Garg S.K., Smith J., Beatson C., Lopez-Baca B., Voelmle M., Gottlieb P.A. (2009). Comparison of accuracy and safety of the SEVEN and the Navigator continuous glucose monitoring systems. Diabetes Technol. Ther..

[13] Luijf Y.M., Avogaro A., Benesch C., Bruttomesso D., Cobelli C., Ellmerer M., Heinemann L., Mader J.K., DeVries J.H. (2012). Continuous glucose monitoring accuracy results vary between assessment at home and assessment at the clinical research center. J. Diabetes Sci. Technol..

[14] Luijf Y.M., Mader J.K., Doll W., Pieber T., Farret A., Place J., Renard E., Bruttomesso D., Filippi A., Avogaro A. (2013). Accuracy and reliability of continuous glucose monitoring systems: A head-to-head comparison. Diabetes Technol. Ther..

[15] Freckmann G., Pleus S., Link M., Zschornack E., Klötzer H.M., Haug C. (2013). Performance Evaluation of Three Continuous Glucose Monitoring Systems: Comparison of Six Sensors per Subject in Parallel. J. Diabetes Sci. Technol..

[16] Leelarathna L., Nodale M., Allen J.M., Elleri D., Kumareswaran K., Haidar A., Caldwell K., Wilinska M.E., Acerini C.L., Evans M.L. (2013). Evaluating the accuracy and large inaccuracy of two continuous glucose monitoring systems. Diabetes Technol. Ther..

[17] Pleus S., Schmid C., Link M., Zschornack E., Klötzer H.M., Haug C., Freckmann G. (2013). Performance evaluation of a continuous glucose monitoring system under conditions similar to daily life. J. Diabetes Sci. Technol..

[18] Damiano E.R., El-Khatib F.H., Zheng H., Nathan D.M., Russell S.J. (2013). A comparative effectiveness analysis of three continuous glucose monitors. Diabetes Care.

[19] Damiano E.R., McKeon K., El-Khatib F.H., Zheng H., Nathan D.M., Russell S.J. (2014). A comparative effectiveness analysis of three continuous glucose monitors: The Navigator, G4 Platinum, and Enlite. J. Diabetes Sci. Technol..

[20] Bailey T.S., Ahmann A., Brazg R., Christiansen M., Garg S., Watkins E., Welsh J.B., Lee S.W. (2014). Accuracy and acceptability of the 6-day Enlite continuous subcutaneous glucose sensor. Diabetes Technol. Ther..

[21] Bailey T., Bode B.W., Christiansen M.P., Klaff L.J., Alva S. (2015). The performance and usability of a factory-calibrated flash glucose monitoring system. Diabetes Technol. Ther..

[22] Kropff J., Bruttomesso D., Doll W., Farret A., Galasso S., Luijf Y.M., Mader J.K., Place J., Boscari F., Pieber T. (2015). Accuracy of two continuous glucose monitoring systems: A head-to-head comparison under clinical research centre and daily life conditions. Diabetes Obes. Metab..

[23] Bonora B., Maran A., Ciciliot S., Avogaro A., Fadini G. (2016). Head-to-head comparison between flash and continuous glucose monitoring systems in outpatients with type 1 diabetes. J. Endocrinol. Investig..

[24] Laffel L. (2016). Improved accuracy of continuous glucose monitoring systems in pediatric patients with diabetes mellitus: results from two studies. Diabetes Technol. Ther..

[25] Aberer F., Hajnsek M., Rumpler M., Zenz S., Baumann P.M., Elsayed H., Puffing A., Treiber G., Pieber T.R., Sourij H. (2017). Evaluation of subcutaneous glucose monitoring systems under routine environmental conditions in patients with type 1 diabetes. Diabetes Obes. Metab..

[26] Kropff J., Choudhary P., Neupane S., Barnard K., Bain S.C., Kapitza C., Forst T., Link M., Dehennis A., DeVries J.H. (2017). Accuracy and longevity of an implantable continuous glucose sensor in the PRECISE study: A 180-day, prospective, multicenter, pivotal trial. Diabetes Care.

[27] Kirchsteiger H., Heinemann L., Freckmann G., Lodwig V., Schmelzeisen-Redeker G., Schoemaker M., del Re L. (2015). Performance comparison of CGM systems: MARD values are not always a reliable indicator of CGM system accuracy. J. Diabetes Sci. Technol..

[28] Reiterer F., Polterauer P., Schoemaker M., Schmelzeisen-Redecker G., Freckmann G., Heinemann L., del Re L. (2017). Significance and Reliability of MARD for the Accuracy of CGM Systems. J. Diabetes Sci. Technol..

[29] Ajjan R.A., Cummings M.H., Jennings P., Leelarathna L., Rayman G., Wilmot E.G. (2018). Accuracy of flash glucose monitoring and continuous glucose monitoring technologies: Implications for clinical practice. Diabetes Vasc. Dis. Res..

[30] Oliver N., Toumazou C., Cass A., Johnston D. (2009). Glucose sensors: A review of current and emerging technology. Diabet. Med..

[31] Vaddiraju S., Burgess D.J., Tomazos I., Jain F.C., Papadimitrakopoulos F. (2010). Technologies for continuous glucose monitoring: Current problems and future promises. J. Diabetes Sci. Technol..

[32] Zarkogianni K., Litsa E., Mitsis K., Wu P.Y., Kaddi C.D., Cheng C.W., Wang M.D., Nikita K.S. (2015). A review of emerging technologies for the management of diabetes mellitus. IEEE Trans. Biomed. Eng..

[33] Lodwig V., Heinemann L. (2003). Continuous glucose monitoring with glucose sensors: Calibration and assessment criteria. Diabetes Technol. Ther..

[34] Bequette B.W. (2010). Continuous Glucose Monitoring: Real-Time Algorithms for Calibration, Filtering, and Alarms. J. Diabete.

[35] Lee J.B., Dassau E., Doyle F.J. A Run-to-Run Approach to Enhance Continuous Glucose Monitor Accuracy Based on Continuous Wear. Proceedings of the 9th IFAC Symposium on Biological and Medical Systems (BMS).

[36] Barcelo-Rico F., Diez J.L., Rossetti P., Vehi J., Bondia J. (2013). Adaptive calibration algorithm for plasma glucose estimation in continuous glucose monitoring. IEEE J. Biomed. Health Inform..

[37] Facchinetti A., Sparacino G., Guerra S., Luijf Y.M., DeVries J.H., Mader J.K., Ellmerer M., Benesch C., Heinemann L., Bruttomesso D. (2013). Real-time improvement of continuous glucose monitoring accuracy. Diabetes Care.

[38] Mahmoudi Z., Dencker Johansen M., Christiansen J.S., Hejlesen O.K. (2013). A multistep algorithm for processing and calibration of microdialysis continuous glucose monitoring data. Diabetes Technol. Ther..

[39] Kirchsteiger H., Zaccarian L., Renard E., del Re L. (2015). LMI-based approaches for the calibration of continuous glucose measurement sensors. IEEE J. Biomed. Health Inform..

[40] Vettoretti M., Facchinetti A., Del Favero S., Sparacino G., Cobelli C. (2016). Online calibration of glucose sensors from the measured current by a time-varying calibration function and Bayesian priors. IEEE Trans. Biomed. Eng..

[41] Acciaroli G., Vettoretti M., Facchinetti A., Sparacino G., Cobelli C. (2018). Reduction of Blood Glucose Measurements to Calibrate Subcutaneous Glucose Sensors: A Bayesian Multiday Framework. IEEE Trans. Biomed. Eng..

[42] Twomey P. (2004). Plasma glucose measurement with the Yellow Springs Glucose 2300 STAT and the Olympus AU640. J. Clin. Pathol..

[43] Clinical and Laboratory Standards Institute (2008). Performance Metrics for Continuous Interstitial Glucose Monitoring.

[44] Del Favero S., Facchinetti A., Sparacino G., Cobelli C. (2014). Improving Accuracy and Precision of Glucose Sensor Profiles: Retrospective Fitting by Constrained Deconvolution. IEEE Trans. Biomed. Eng..

[45] Rebrin K., Steil G., Van Antwerp W., Mastrototaro J. (1999). Subcutaneous glucose predicts plasma glucose independent of insulin: implications for continuous monitoring. Am. J. Physiol. Endocrinol. Metab..

[46] Schiavon M., Dalla Man C., Dube S., Slama M., Kudva Y.C., Peyser T., Basu A., Basu R., Cobelli C. (2015). Modeling plasma-to-interstitium glucose kinetics from multitracer plasma and microdialysis data. Diabetes Technol. Ther..

[47] Huyett L.M., Dassau E., Zisser H.C., Doyle F.J. The impact of glucose sensing dynamics on the closed-loop artificial pancreas. Proceedings of the 2015 American Control Conference (ACC).

[48] Guerra S., Facchinetti A., Sparacino G., De Nicolao G., Cobelli C. (2012). Enhancing the Accuracy of Subcutaneous Glucose Sensors: A Real-Time Deconvolution-Based Approach. IEEE Trans. Biomed. Eng..

[49] Schmelzeisen-Redeker G., Schoemaker M., Kirchsteiger H., Freckmann G., Heinemann L., del Re L. (2015). Time delay of CGM sensors: Relevance, causes, and countermeasures. J. Diabetes Sci. Technol..

[50] Schmelzeisen-Redeker G., Staib A., Strasser M., Müller U., Schoemaker M. (2013). Overview of a Novel Sensor for Continuous Glucose Monitoring. J. Diabetes Sci. Technol..

[51] Campos-Náñez E., Breton M.D. (2017). Effect of BGM Accuracy on the Clinical Performance of CGM: An In-Silico Study. J. Diabetes Sci. Technol..

[52] Banauch D., Brümmer W., Ebeling W., Metz H., Rindfrey H., Lang H., Leybold K., Rick W. (1975). Eine glucose-dehydrogenase für die glucose-bestimmung in körperflüssigkeiten. Clin. Chem. Lab. Med..

[53] Andelin M., Kropff J., Matuleviciene V., Joseph J.I., Attvall S., Theodorsson E., Hirsch I.B., Imberg H., Dahlqvist S., Klonoff D. (2016). Assessing the accuracy of continuous glucose monitoring (CGM) calibrated with capillary values using capillary or venous glucose levels as a reference. J. Diabetes Sci. Technol..

[54] Rodbard D. (2014). Characterizing accuracy and precision of glucose sensors and meters. J. Diabetes Sci. Technol..

[55] Vettoretti M., Facchinetti A., Sparacino G., Cobelli C. (2017). A Model of Self-Monitoring Blood Glucose Measurement Error. J. Diabetes Sci. Technol..

[56] Facchinetti A., Del Favero S., Sparacino G., Castle J., Ward W., Cobelli C. (2014). Modeling the Glucose Sensor Error. IEEE Trans. Biomed. Eng..

[57] Facchinetti A., Favero S.D., Sparacino G., Cobelli C. (2015). Model of glucose sensor error components: Identification and assessment for new Dexcom G4 generation devices. Med. Biol. Eng. Comput..

[58] Reiterer F., Polterauer P., Freckmann G., del Re L. Identification of CGM Time Delays and Implications for BG Control in T1DM. Proceedings of the XIV Mediterranean Conference on Medical and Biological Engineering and Computing (Medicon 2016).

[59] Pleus S., Schoemaker M., Morgenstern K., Schmelzeisen-Redeker G., Haug C., Link M., Zschornack E., Freckmann G. (2015). Rate-of-change dependence of the performance of two CGM systems during induced glucose swings. J. Diabetes Sci. Technol..

[60] Meeker W.Q., Hahn G.J., Escobar L.A. (2017). Statistical Intervals: A Guide for Practitioners and Researchers.

[61] Johnson R., Wichern D. (2007). Applied Multivariate Statistical Analysis.

[62] Hoffman K. (2007). Banach Spaces of Analytic Functions.

[63] Scott D.W. (2015). Multivariate Density Estimation: Theory, Practice, and Visualization.

[64] Silverman B. (1986). Density Estimation for Statistics and Data Analysis.

